# Younger Age Is Associated with Poorer Survival in Patients with Signet-Ring Cell Carcinoma of the Colon without Distant Metastasis

**DOI:** 10.1155/2016/2913493

**Published:** 2016-11-22

**Authors:** Ben Huang, Mengdong Ni, Chen Chen, Yang Feng, Sanjun Cai

**Affiliations:** Department of Colorectal Surgery, Fudan University Shanghai Cancer Center, 270 Dong'an Road, Shanghai 20032, China

## Abstract

*Background*. In general, younger age is associated with better survival in patients with colon cancer. In this study, we aim to analyze the impact of age on cancer-specific survival (CSS) in patients with signet-ring cell carcinoma (SRCC) of the colon, a particularly aggressive type of colon cancer.* Methods*. Information on patients with SRCC of the colon with no distant metastasis was extracted from the US Surveillance, Epidemiology, and End Results (SEER) database. An X-tile plot was used to determine the optimal cutoff age at diagnosis.* Results*. A total of 776 patients were included in data analysis. The X-tile program revealed an optimal cutoff at 35 years of age. A higher percentage of stage III disease and a higher percentage of N2 disease were observed in patients ≤ 35 years of age. The multivariate Cox proportional model demonstrated that patients ≤ 35 years of age were more likely to have a poorer survival outcome compared with patients aged >35 years (HR 1.411, 95% CI 1.032–1.929, and *P* = 0.031).* Conclusion*. In contrast to the association of younger age with better survival in colon cancer patients, younger age (≤35 years) is associated with poorer survival outcome in patients with SRCC of the colon without distant metastasis.

## 1. Introduction

Signet-ring cell carcinoma (SRCC) is a rare but distinct subtype of colon cancer and makes up roughly 1% of all colorectal cancer cases [1–5]. It is a very aggressive form of colon cancer [1, 6] and is associated with poor biological behaviors, including poor differentiation [1, 2], perineural or lymphovascular invasion [1, 7], and lymph node involvement [4, 8]. From a molecular biological perspective, colorectal SRCC is also recognized as a unique tumor entity, inclining to have high levels of* BRAF* mutations,* KRAS* mutations, and CpG island methylation [7, 9, 10].

The incidence rate of colorectal cancer has been decreasing over the past three decades [11], mostly due to the decline in adenocarcinoma [12]. By contrast, the incidence rate of SRCC has slightly increased over the past few years, probably due to increasing recognition of the disease in daily practice [12]. Hitherto, the prognostic determinants of SRCC remain largely undefined. Lee et al. [13] studied 19 patients with primary SRCC of the colon and rectum who underwent curative surgery and found that these patients had a poorer prognosis compared to other types of colorectal cancer. Belli et al. [14] reported a retrospective series of 22 colorectal SRCC patients and they found that these patients were diagnosed at an advanced stage and that, among others, tumor site and TNM stage had significant effect on survival.

Colorectal SRCC is noted for a significantly higher proportion of younger patients compared to non-SRCC colorectal cancer. A large population-based study, including 196,757 cases of colorectal cancer, showed that 7.7% of SRCC patients were under the age of 45 years, whereas only 2.7% of colorectal adenocarcinoma patients were younger than 45 years of age [3]. Li et al. [15] evaluated 69,835 patients with colorectal cancer in the Surveillance, Epidemiology, and End Results (SEER) database and found a significantly higher rate of SRCC in patients younger than 40 years of age (2.8%) than those older than 40 years of age (0.8%).

For patients with colorectal cancer in general, younger age is associated with better or comparable survival [15–19]. Currently, scant knowledge is available on the prognostic value of younger age in SRCC of the colon. Considering the aggressive biological behavior and extremely poor oncologic outcomes of SRCC of the colon, we hypothesized that younger patients with this particular histological subtype of colon cancer may harbor a biologically aggressive phenotype and have worse prognosis than older patients. The SEER database contains 18 cancer registries covering 26% of the US population, collecting and providing cancer incidence and survival data. To address this hypothesis, we analyzed the cancer-specific survival (CSS) of patients with SRCC of the colon without distant metastasis in the SEER database and determined the prognostic value of age and other variables for CSS. Subgroup analysis was conducted in stage I/II and stage III SRCC of the colon.

## 2. Materials and Methods

### 2.1. Patient Selection

We extracted the demographic and clinicopathological records of invasive colon cancer patients from January 1988 to December 2011 from the SEER database (http://seer.cancer.gov/, April 2013 release). Patients meeting the following criteria were included in the current analysis: (1) age between 18 and 74 years at the time of diagnosis; (2) pathologically confirmed SRCC of the colon; (3) known depth of invasion and lymph node status; (4) at least 12 lymph nodes harvested; (5) colon cancer surgically resected with pathology specimen; (6) known survival time and cause of death. Patients were excluded if (1) they underwent only local tumor excision; (2) diagnosis of colon cancer was obtained from death certificate or by autopsy; (3) there was distant metastasis of colon cancer (AJCC stage M0); (4) there were other concurrent malignancies. The study protocol was reviewed and approved by the Fudan University Shanghai Cancer Center Ethical Committee and Institutional Review Board. Informed consent is not applicable since the study was based on a publicly available database (the SEER database).

### 2.2. Outcome Measures

Data on the following variables were retrieved from the SEER database: gender, race, age at diagnosis, years of diagnosis, pathological grade, number of primary lesions, number of lymph nodes harvested and positive lymph nodes (N0, N1, and N2), and depth of local invasion (T1, T2, T3, and T4), American Joint Committee on Cancer (AJCC) TNM stage, radiation sequence with surgery, follow-up duration, and SEER cancer-specific death classification. All cases were restaged by the 7th AJCC TNM staging system. In this study, the right colon refers to the cecum, the ascending colon, the hepatic flexure of the colon, and the transverse colon, whereas the left colon refers the splenic flexure of the colon, the descending colon, and the sigmoid colon.

### 2.3. Statistical Analysis

CSS was the primary endpoint of our study and was calculated from the time of diagnosis to the time of colon cancer-specific death. Patients who died from other causes or were alive at the last follow-up were censored. We employed the X-tile software (http://medicine.yale.edu/lab/rimm/research/software.aspx) (Yale School of Medicine, CT, USA) to determine the optimal cutoff age at diagnosis using the minimum *P* values from log-rank chi-squared statistics for stratification of patients into the high or low risk group [20]. The X-tile plots allow a single, global assessment of every possible way of dividing a population into low and high risk for survival. In the X-tile analysis, data are displayed in the *x*-axis where each point reflects a different cutoff point. The intensity of color in the grid indicates the strength of association, in this study, between age at diagnosis and CSS. Patient data stratified by the cutoff age were summarized using cross-tabulation, and the distributions were compared using chi-squared tests.

Survival curves were plotted using the Kaplan-Meier method. In addition, log-rank test was used for univariate analysis and variables with a *P* value < 0.1 were entered into the Cox proportion hazard regression model. Multivariate Cox regression analyses were used to generate adjusted hazards ratios (HR) and their corresponding 95% confidence intervals (CIs). Subgroup analyses were conducted in stage I/II and stage III SRCC of the colon. All statistical analyses were performed using SPSS version 20.0 for Windows (SPSS Inc., Chicago, IL, USA). A two-sided *P* value of less than 0.05 was accepted as statistically significant.

## 3. Results

### 3.1. Demographic and Baseline Characteristics of SRCC Patients

Seven hundred seventy-six cases (432 men and 344 women) from the SEER database were included in data analysis. The sample was predominantly Caucasian (82.1%). Patient demographic and clinicopathological features are shown in Table 1. Their mean age at diagnosis was 56 years and 90.0% (698) of them were aged more than 35 years. The median follow-up duration was 27 (interquartile range, 12 to 64) months. The primary lesion was in the right colon in 78.9% of the cases. Most patients (89.7%) had poor or undifferentiated SRCC. Furthermore, 400 (51.6%) subjects had metastasis in more than three lymph nodes (N_2_). TNM stage III cases were the most common, accounting for 74.9% (581), and stage I cases the least common 4.5% (35). Moreover, compared to patients > 35 years of age, patients ≤ 35 years of age had a significantly higher proportion of non-Caucasians (35.9% versus 15.9%, *P* < 0.001) and a markedly greater percentage of N_2_ SRCC (73.1% versus 49.1%, *P* < 0.001) and stage III SRCC (89.7% versus 73.2%, *P* = 0.006) (Table 1).

### 3.2. Impact of Age on Survival of SRCC Patients

**Figure 1:**
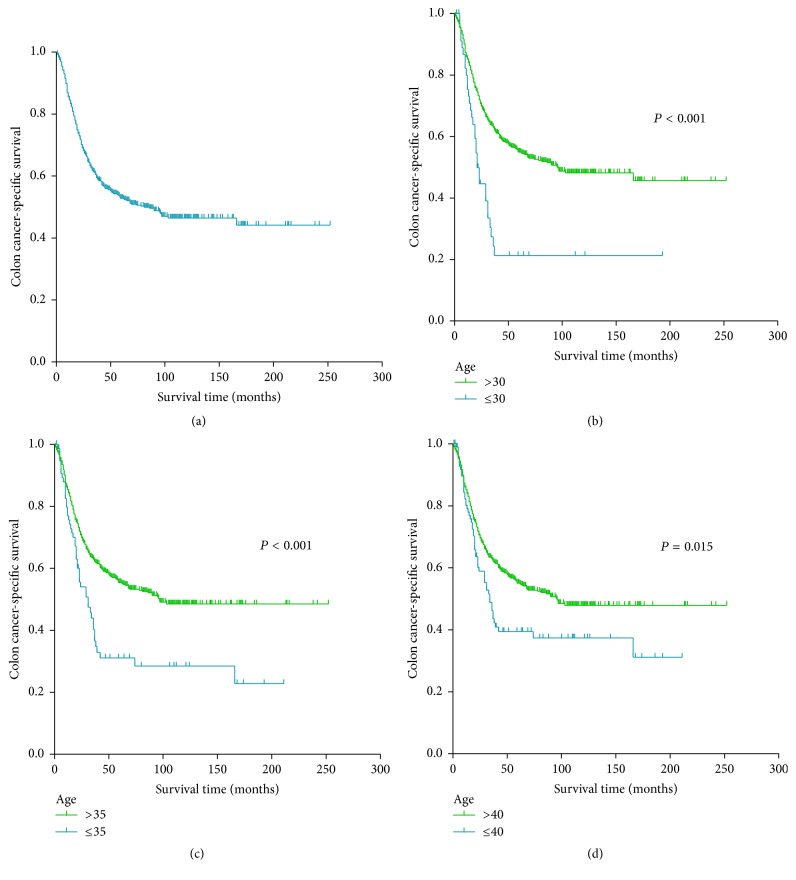
(a) The Kaplan-Meier survival curve for patients with nonmetastatic signet-ring cell carcinoma (SRCC) of the colon from the Surveillance, Epidemiology, and End Results (SEER) cohort. Kaplan-Meier curves for patients with nonmetastatic SRCC of the colon stratified by (b) 30 years of age, (c) 35 years of age, and (d) 40 years of age.

**Figure 2:**
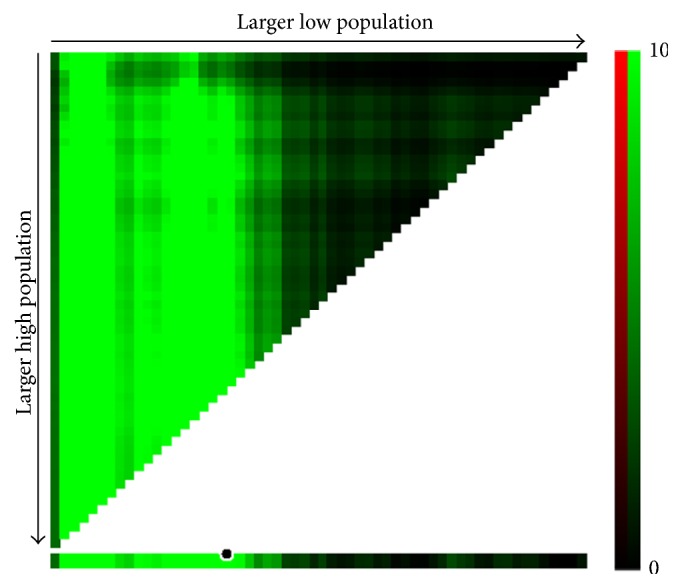
X-tile plot of age at diagnosis in the SEER cohort of patients with SRCC of the colon. *x*-axis represents all age cutoff values from low to high (left to right) to define the high and low subset. Brighter pixels indicate stronger association between age at diagnosis and cancer-specific survival (CSS). The plot shows the brightest pixel (marked by the black circle) when the study cohort is divided into the high and low subset using 35 years of age as the cutoff. Green color indicates continuous direct association between increasing age at diagnosis and greater CSS.

In this study, 318 (41.0%) colon cancer-specific deaths were observed. The one-year CSS stood at 83.8%, the three-year CSS at 59.8%, and the five-year CSS at 52.2% for the entire cohort (Figure 1(a)). Kaplan-Meier analyses showed that younger patients had poorer CSS than older patients (*P* < 0.001 at a cutoff of 30 years of age, Figure 1(b); *P* < 0.001 at a cutoff of 35 years of age, Figure 1(c); *P* = 0.015 at a cutoff of 40 years of age, Figure 1(d)). An X-tile analysis indicated optimal cutoff age at 35 years (Figure 2). When 35 years of age was used as the optimal cutoff to stratify patient survival, the Kaplan-Meier analysis revealed that patients ≤ 35 years of age had poorer CSS compared with patients > 35 years and the 5-year CSS was 31.1% and 54.9% in patients ≤ 35 years and patients > 35 years (*P* < 0.001) (Figure 1(c)). An analysis using the multivariate Cox proportional model further demonstrated that age was an independent prognostic factor for CSS. Patients ≤ 35 years of age were more likely to have a poorer survival outcome compared with patients aged > 35 years (HR 1.411, 95% CI 1.032–1.929, and *P* = 0.031) (Table 3).

Our univariate analysis showed that, apart from age, primary site (*P* = 0.049), N and T stage (*P* < 0.001 in both) were prognostic determinants of CSS (Table 2). Analysis using a multivariate Cox proportional model suggested that, in addition to age at diagnosis, N and T stages were independent determinants of CSS of SRCC patients from the SEER database (*P* < 0.001 in both) (Table 3). Compared to N0 stage patients, N2 stage patients were more than 6 times more likely to succumb to SRCC (HR 6.392, 95% CI 4.102–9.961, and *P* < 0.001). However, multivariate analysis revealed no significant difference between SRCC patients with T1 stage and T4 stage (HR: 3.988; 95% CI: 0.970–16.391, *P* = 0.055).

**Figure 3:**
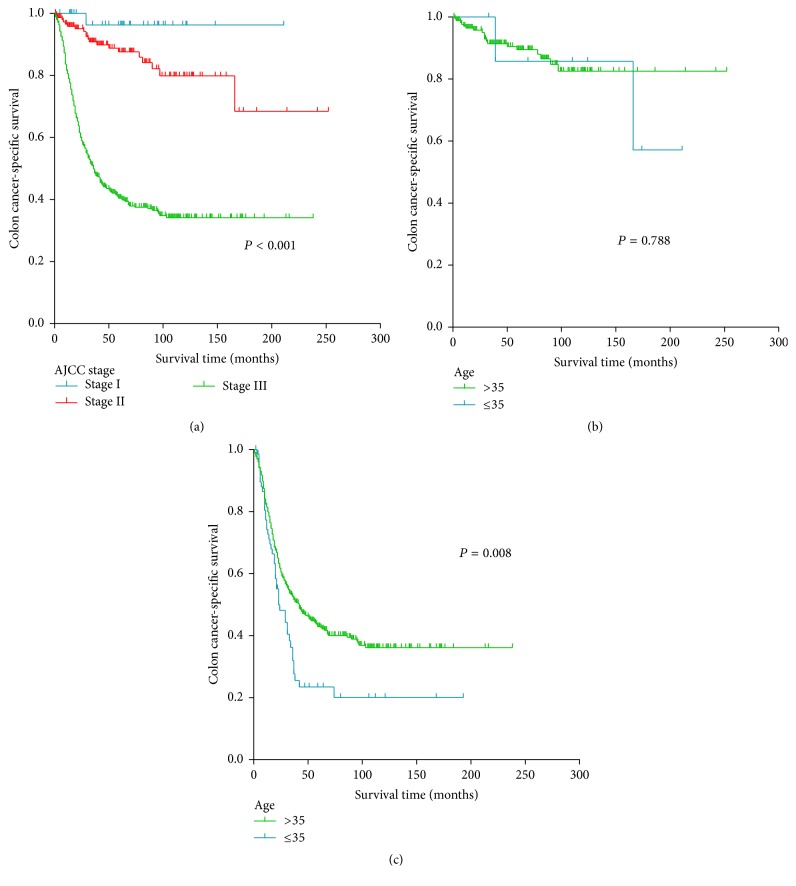
(a) Kaplan-Meier curves for patients with nonmetastatic SRCC of the colon stratified by TNM stage. Kaplan-Meier curves for patients with (b) stage I/II and (c) stage III SRCC of the colon stratified by age at diagnosis (≤35 or >35 years).

Kaplan-Meier curves for stage I, stage II, and stage III SRCC patients were shown in Figure 3(a). In the planned subgroup analysis, the association between young age (35 years as the cutoff) and poor CSS was not observed in patients with stage I/II SRCC (*P* = 0.788) (Figure 3(b)). By contrast, stage III SRCC patients ≤ 35 years of age had a significantly lower CSS compared with those > 35 years (*P* = 0.008), the 5-year CSS was 23.4% and 42.5% in patients ≤ 35 years and patients > 35 years (Figure 3(c)). Multivariate Cox proportional model was further conducted in stage III SRCC patients. Consistently, age (*P* = 0.034), N stage (*P* < 0.001), and T stage (*P* = 0.003) were independent prognostic determinants of CSS for stage III SRCC patients (Table 4). Compared with stage III SRCC >35 years of age, patients ≤ 35 years of age were more likely to have a poorer CSS (HR, 1.416, 95% CI 1.027–1.951, *P* = 0.034) (Table 4).

## 4. Discussion

Colorectal SRCC often presents as advanced tumors [4, 21], with metastases at multiple sites, especially peritoneal carcinomatosis [8, 21–23]. The disease is typically diagnosed at an advanced TNM stage [2–4] and has a dismal clinical outcome. However, the prognostic determinants of colorectal SRCC remain largely undefined. In the current study, we analyzed the demographic and clinicopathological variables of 776 cases of SRCC of the colon without distant metastasis in the SEER database. We demonstrated a five-year CSS of 52.2% for the SRCC cohort from the SEER database. The major finding is poor survival in young patients, with an optimal cutoff at 35 years of age. The most recent studies, however, reported that age showed no correlation with survival in patients with colorectal SRCC. Wang et al. [24] analyzed 59 patients with colorectal SRCC and found that age (40 years old as cutoff value) was not a prognostic factor for survival. Kakar and Smyrk [25] reported that age (70 years old as cutoff value) was not significantly associated with survival in a study including 72 patients with SRCC of the colorectum.

Colorectal SRCC tends to occur in younger patients compared to non-SRCC colorectal cancer. It remains controversial whether younger colorectal cancer patients had comparable or better prognosis versus older patients [15, 18, 19, 26–28]. We found that younger patients with SRCC of the colon without distant metastasis (≤35 years of age) fared poorer in CSS compared with older patients (>35 years of age). A preplanned subgroup analysis confirmed the association of young age with poor outcome in patients with stage III SRCC but not stage I/II disease. Also, a markedly higher percentage of N2 SRCC and a markedly higher percentage of stage III SRCC were observed in younger patients (≤35 years of age). Younger patients may mistakenly believe that they are unlikely to harbor malignant tumors and consequently seek medical attention at a more advanced stage of SRCC. Equally likely, physicians may miss malignancy in younger SRCC patients due to lowered suspicion, thus missing the best time of treatment. These phenomena may partially contribute to our findings. Also, the late occurrence of clinical symptoms in patients with colorectal SRCC may lead to delay in diagnosis [29, 30].

The unfathomed distinct genetic basis of young-onset SRCC of the colon may contribute to the poor survival outcomes. As a subtype of colorectal cancer with poor survival, SRCC has intrinsic genetic events that are responsible for its highly malignant nature [1]. Colorectal SRCC has more frequent* BRAF* mutations and MLH1 loss, less frequent 18q LOH, and lower COX2 levels [31], when compared with non-SRCC colorectal cancer. MSI-H status is closely associated with signet-ring cell differentiation with rates ranging from 43 to 86% in different studies [32–34]; however, no recognized rate has been reported yet due to small sample size. Likewise, young-onset colorectal cancer also has distinct genetic basis. Many studies have reported a higher frequency of MSI positive colorectal cancers in very young patients [35–37]. Morris et al. [35] showed that, besides MSI, colorectal cancer in young patients correlated well with various molecular features of tumors, such as higher rates of* TP-53* mutation and lower rates of* BRAF* and* KRAS* mutations. However, the exact genetic features of young-onset SRCC of the colon have been rarely reported. A small number of reports of genetic changes in young colorectal SRCC patients and young patients with SRCC in other body sites such as the stomach are available [38–41]; therefore, further comparative studies of larger patient sample sizes are needed to delineate the genetic peculiarity of young-onset SRCC of the colon.

Based on the data from the SEER database, our study explores the prognostic role of age in determining survival outcomes of patients with SRCC of the colon. Since SRCC is a rare histological cancer subtype, current knowledge was mostly derived from single institution studies based on small population sizes [6, 23]. We utilized the SEER database to ensure a large sample size, and, to be specific, our study included a total of 776 patients. However, there are still several limitations in our study. Although SEER is a large population-based database, the stratification by tumor stage reduced the number of subjects in each study group and statistical power. One remarkable limitation is that the homogeneity of our study population may be impaired by the long study period because there have been rapid developments in colon cancer screening, diagnosing, imaging, and treating during the years from 1988 to 2011. An additional limitation is that some important patient- and disease-related information cannot be obtained from the SEER database, such as intestinal obstruction or penetration, comorbidities, surgical margin status, and data on adjuvant chemotherapy. Furthermore, the SEER database does not have records on family history or molecular biology information; however, such information may be a valuable addition to current data and may help clarify and further understand the risk factors for young-onset SRCC of the colon.

## 5. Conclusion

In conclusion, our analysis indicated that, in contrast to the association of younger age with better survival in colon cancer patients, younger age is associated with a worse survival in patients with SRCC of the colon without distant metastasis, particularly in patients with stage III disease. Further studies are warranted to uncover potential molecular and genetic characteristics of young patients with SRCC of the colon.

## Figures and Tables

**Table 1:** Demographic and baseline characteristics of patients with nonmetastatic signet-ring cell carcinoma of the colon from the SEER database and stratification by age at diagnosis.

Characteristics	All patients	≤35 years	>35 years	*P* value (Pearson *χ* ^2^)
*N* (%)	776 (100.0)	78 (10.0)	698 (90.0)	
Median follow-up, months	27	23	28	
Male gender, *n* (%)	432 (55.7)	42 (53.8)	390 (55.9)	0.732 (0.12)
Years of diagnosis, *n* (%)				0.029 (4.76)
1988–2003	309 (39.8)	40 (51.3)	269 (38.5)	
2004–2011	467 (60.2)	38 (48.7)	429 (61.5)	
Primary site, *n* (%)				0.001 (10.31)
Right colon	612 (78.9)	50 (64.1)	562 (80.5)	
Left colon	164 (21.1)	28 (35.9)	136 (19.5)	
Race, *n* (%)				<0.001 (19.84)
White	637 (82.1)	50 (64.1)	587 (84.1)	
Black	83 (10.7)	16 (20.5)	67 (9.6)	
Others^a^	56 (7.1)	12 (15.4)	44 (6.3)	
Pathology grade, *n* (%)				0.987 (0.001)
Well/moderate	80 (10.3)	8 (10.3)	72 (10.3)	
Poor/undifferentiated	696 (89.7)	70 (89.7)	626 (89.7)	
Tumor size, *n* (%)				0.123 (2.49)
≤4.0 cm	249 (32.1)	19 (24.4)	230 (33.0)	
>4.0 cm	527 (67.9)	59 (75.6)	468 (67.0)	
LNH, *n* (%)				0.023 (5.19)
≤18	343 (44.2)	25 (32.1)	318 (45.6)	
>18	433 (55.8)	53 (67.9)	380 (54.4)	
T stage, *n* (%)				0.164 (5.11)
T1	24 (3.1)	2 (2.5)	22 (3.1)	
T2	31 (4.0)	1 (1.3)	30 (4.3)	
T3	453 (58.4)	40 (51.3)	413 (59.2)	
T4	268 (34.5)	35 (44.9)	233 (33.4)	
N stage, *n* (%)				<0.001 (17.08)
N0	195 (25.1)	8 (10.2)	187 (26.8)	
N1	181 (23.3)	13 (16.7)	168 (24.1)	
N2	400 (51.6)	57 (73.1)	343 (49.1)	
TNM stage, *n* (%)				0.006 (10.27)
I	35 (4.5)	1 (1.3)	34 (4.9)	
II	160 (20.6)	7 (9.0)	153 (21.9)	
III	581 (74.9)	70 (89.7)	511 (73.2)	

LNH: number of lymph nodes harvested.

^a^Native Americans, Asians, Pacific Islanders, and unknown.

**Table 2:** Univariate analyses of determinants of cancer-specific survival (CSS) of patients with nonmetastatic signet-ring cell carcinoma of the colon.

Variable	5-year CSS	Log rank *χ* ^2^	*P* value
Sex		0.466	0.495
Male	51.5%		
Female	53.4%		
Years of diagnosis		1.801	0.180
1988–2003	50.5%		
2004–2011	53.7%		
Race		3.169	0.205
White	53.7%		
Black	43.4%		
Other^a^	50.2%		
Pathology grade		1.503	0.138
Well/moderate	58.4%		
Poor/undifferentiated	52.5%		
LNH		0.168	0.682
≤18	51.2%		
>18	53.5%		
Tumor size (cm)		1.816	0.178
≤4.0	54.8%		
>4.0	51.9%		
Primary site		3.859	0.049
Right colon	54.9%		
Left colon	43.3%		
Age at diagnosis (year)		13.121	<0.001
≤35	31.1%		
>35	54.9%		
N stage		148.599	<0.001
N0	88.3%		
N1	61.2%		
N2	30.0%		
T stage		63.528	<0.001
T1	90.4%		
T2	84.1%		
T3	59.8%		
T4	30.2%		

LNH: number of lymph nodes harvested.

^a^Includes Native American, Asian, Pacific Islander, and unknown.

**Table 3:** Multivariate analyses of determinants of cancer-specific survival (CSS) of patients with nonmetastatic signet-ring cell carcinoma of the colon.

Variables	Multivariate analysis		
	HR	95% CI	*P* value
Primary site			0.696
Right colon	1	Reference	
Left colon	0.950	0.732–1.231	
Age at diagnosis (year)			0.031
>35	1	Reference	
≤35	1.411	1.032–1.929	
N stage			<0.001
N0	1	Reference	
N1	2.977	1.840–4.817	<0.001
N2	6.392	4.102–9.961	<0.001
T stage			<0.001
T1	1	Reference	
T2	1.406	0.257–7.707	0.695
T3	2.458	0.602–10.042	0.210
T4	3.988	0.970–16.391	0.055

HR: hazard ratio; CI: confidence interval.

**Table 4:** Multivariate survival analyses of determinants of cancer-specific survival (CSS) of patients with stage III signet-ring cell carcinoma of the colon.

Variables	Multivariate analysis		
	HR	95% CI	*P* value
Primary site			0.651
Right colon	1	Reference	
Left colon	0.940	0.721–1.227	
Age at diagnosis (year)			0.034
>35	1	Reference	
≤35	1.416	1.027–1.951	
N stage			<0.001
N1	1	Reference	
N2	2.170	1.628–2.894	
T stage			0.003
T1	1	Reference	
T2	1.438	0.149–13.830	0.753
T3	2.472	0.345–17.723	0.368
T4	3.721	0.518–26.753	0.192

HR: hazards ratio; CI: confidence interval.
